# Tumor Nonimmune-Microenvironment-Related Gene Expression Signature Predicts Brain Metastasis in Lung Adenocarcinoma Patients after Surgery: A Machine Learning Approach Using Gene Expression Profiling

**DOI:** 10.3390/cancers13174468

**Published:** 2021-09-05

**Authors:** Seokjin Haam, Jae-Ho Han, Hyun Woo Lee, Young Wha Koh

**Affiliations:** 1 Department of Thoracic and Cardiovascular Surgery, Ajou University School of Medicine, Suwon 16499, Korea; haamsj@aumc.ac.kr; 2 Department of Pathology, Ajou University School of Medicine, Suwon 16499, Korea; hanpathol@naver.com; 3 Department of Hematology-Oncology, Ajou University School of Medicine, Suwon 16499, Korea; leehw@ajou.ac.kr

**Keywords:** lung adenocarcinoma, brain metastasis, gene expression profile, tumor nonimmune microenvironment, extracellular matrix, machine learning

## Abstract

**Simple Summary:**

It is important to be able to predict brain metastasis in lung adenocarcinoma patients; however, research in this area is still lacking. Much of the previous work on tumor microenvironments in lung adenocarcinoma with brain metastasis concerns the tumor immune microenvironment. The importance of the tumor nonimmune microenvironment (extracellular matrix (ECM), epithelial–mesenchymal transition (EMT) feature, and angiogenesis) has been overlooked with regard to brain metastasis. We evaluated tumor nonimmune-microenvironment-related gene expression signatures that could predict brain metastasis after the surgical resection of lung adenocarcinoma using a machine learning approach. We identified a tumor nonimmune-microenvironment-related 17-gene expression signature, and this signature showed high brain metastasis predictive power in four machine learning classifiers. The immunohistochemical expression of the top three genes of the 17-gene expression signature yielded similar results to NanoString tests. Our tumor nonimmune-microenvironment-related gene expression signatures are important biological markers that can predict brain metastasis and provide patient-specific treatment options.

**Abstract:**

Using a machine learning approach with a gene expression profile, we discovered a tumor nonimmune-microenvironment-related gene expression signature, including extracellular matrix (ECM) remodeling, epithelial–mesenchymal transition (EMT), and angiogenesis, that could predict brain metastasis (BM) after the surgical resection of 64 lung adenocarcinomas (LUAD). Gene expression profiling identified a tumor nonimmune-microenvironment-related 17-gene expression signature that significantly correlated with BM. Of the 17 genes, 11 were ECM-remodeling-related genes. The 17-gene expression signature showed high BM predictive power in four machine learning classifiers (areas under the receiver operating characteristic curve = 0.845 for naïve Bayes, 0.849 for support vector machine, 0.858 for random forest, and 0.839 for neural network). Subgroup analysis revealed that the BM predictive power of the 17-gene signature was higher in the early-stage LUAD than in the late-stage LUAD. Pathway enrichment analysis showed that the upregulated differentially expressed genes were mainly enriched in the ECM–receptor interaction pathway. The immunohistochemical expression of the top three genes of the 17-gene expression signature yielded similar results to NanoString tests. The tumor nonimmune-microenvironment-related gene expression signatures found in this study are important biological markers that can predict BM and provide patient-specific treatment options.

## 1. Introduction

Lung adenocarcinoma (LUAD) is the most common non-small-cell lung cancer (NSCLC) and is known to cause frequent brain metastasis (BM) [1,2]. In particular, BM is more frequent when oncogenic mutations or structural variations, such as EGFR, ALK, and RET, are found in LUAD [1,2]. The incidence of BM is also increasing as survival time is increasing with the use of targeted agents and immune checkpoint blockers in LUAD. However, in the case of NSCLC with BM, the median overall survival time of treated patients is 4–15 weeks, the survival rate is very low, and complications are very serious [3]. Prophylactic cranial irradiation causes side effects, such as acute encephalopathy or radiation necrosis; therefore, it is difficult to target all patients with NSCLC [4]. Although the gene alterations of EGFR, ALK, and RET frequently induce BM, the number of patients with this mutation is small, and the effectiveness of targeted therapy in BM has not been verified.

Extracellular matrix (ECM) remodeling, epithelial–mesenchymal transition (EMT), and angiogenesis are all closely related to the tumor nonimmune microenvironment and they are well-known mechanisms for the metastasis of primary tumors. Specific alterations in ECM remodeling, EMT, and angiogenesis are also known to trigger BM.

ECM remodeling plays an essential role in the migration and invasion of tumor cells, and eventually plays a role in promoting metastasis [5]. The overexpression of MMP-1 in breast cancer plays an important role in promoting the migration of tumor cells to the endothelium of the brain by breaking down inter-endothelial junctions and disrupting the endothelial integrity [6]. The ECM protein nephronectin promotes breast cancer BM through the integrin-binding domain [7]. A recent study has shown that αv integrin is involved in the adhesion and migration of tumor cells to brain capillaries, thereby promoting BM in lung cancer [8].

EMT is a mechanism by which differentiated epithelial cells change into mesenchymal phenotypes through the loss of cell–cell junctions and the loss of cell polarity [9]. A recent study examined EMT markers in BM samples from primary lung, breast, colon, and kidney tumors and reported an increased expression of TWIST, a representative EMT marker [10]. Cell adhesion molecule 2 (CADM2) induces BM by activating the EMT pathway in NSCLC [11]. SNORA71B, a type of SnoRNA, promotes the migration of breast cancer cells across the blood–brain barrier by activating the EMT pathway [12]. According to a previous study, the inhibition of EMT by cucurbitacin B, an HER2 target therapeutic substance, results in the inhibition of BM in breast cancer cells [13].

Angiogenesis is essential to providing a pathway for metastatic tumor cell migration [14]. Angiogenesis is co-ordinated by proangiogenic or antiangiogenic factors, and is primarily induced by vascular endothelial growth factor (VEGF), one of the most important factors in this process [15]. Integrin αvβ3, a proangiogenic factor, induces BM in breast cancer, and angiogenesis through VEGF activation has also been observed [16]. Angiopoietin-2 has been shown to cause BM by causing blood–brain barrier impairment in a breast cancer model [17]. In a lung cancer model, ADAM9 has been reported to promote angiogenesis by activating VEGF, which subsequently results in BM [18,19].

The machine learning method has been widely used in biomarker discovery in recent years [20,21]. Machine learning is a mathematical algorithm that trains a model on a training data set and applies the model to a test data set [22]. Machine learning consists of classification and feature selection. Classification is a supervised learning process of categorizing a given set of data into classes. The classification model predicts the label of a sample from its features. In our case, the label of the sample was the presence of brain metastases, and the feature was a specific gene expression. Feature selection is the process of removing noise or noninformative features [23]. In our case, it was the process of removing the genes that are less predictive of BM.

We used four machine learning classifiers for the gene expression analysis (naïve Bayes method (NB), neural network (NN), random forest (RF), and support vector machine (SVM)). NN is a machine learning model inspired by the structure and function of biological neural networks [24]. The basic building blocks of NN are artificial neurons. When an artificial neuron receives a signal, it transmits a signal to other nearby artificial neurons. Each connection is assigned a weight indicating its relative importance. Artificial neurons consist of three layers. NN has emerged as a promising machine learning method that is widely used for gene expression analysis [25,26]. NB is based on Bayes’ theorem and is used for solving classification problems [27]. NB assumes that each input feature is independent. NB is also widely used in gene expression analysis [28,29]. RF constructs a multitude of decision trees at training time for classification or regression [30]. It is a popular ensemble learning method in pattern recognition, including gene expression analysis [31,32]. SVM is a supervised learning model and has been widely applied to gene expression analysis [33]. SVM can be used for classification or regression by constructing hyperplanes, or sets of hyperplanes, in a high-dimensional or infinite-dimensional space [34].

Much of the previous work on LUAD BM concerns the oncogenes of the tumor or the immune system of the tumor microenvironment. BM occurs frequently in LUAD with EGFR or K-RAS mutation [35], and specific immune gene expression signatures are frequently found in NSCLC patients with BM [36,37]. Gene expression profiles related to ECM remodeling, EMT, and angiogenesis are likely to predict LUAD BM; however, little research has been conducted. In 64 patients with LUAD, we performed gene expression analysis in patients with BM, and in tumor-node-metastasis *(*TNM) stage-matched patients without BM during postoperative follow-up. A total of 770 genes related to ECM remodeling, EMT, and angiogenesis were evaluated. A machine learning approach was used to verify whether gene expression could predict BM. Whether the predictive ability differed according to the TNM stage was verified through subgroup analysis. A pathway enrichment assay was performed to identify the major pathways leading to LUAD BM, and the results were examined using immunohistochemistry (IHC).

## 2. Results

### 2.1. Prediction Modeling Using Machine Learning Methods

**Figure 1:**
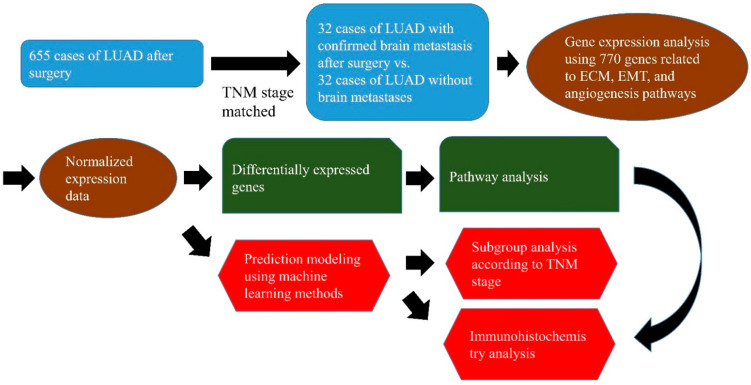
Overview of the workflow of the development of extracellular-matrix-remodeling-related, epithelial–mesenchymal-transition-related, and angiogenesis-related gene signatures that predict the response to brain metastasis in lung adenocarcinoma. ECM, extracellular matrix; EMT, epithelial mesenchymal transition, IHC, immunohistochemistry; LUAD, lung adenocarcinoma.

**Figure 2:**
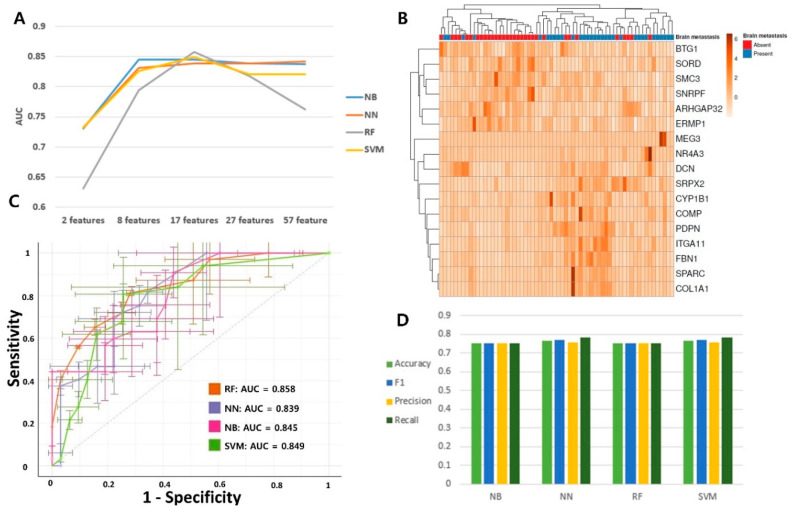
Predictive model building for brain metastasis in lung adenocarcinoma. (**A**) Comparison of areas under the curves (AUC) according to four machine learning algorithms and feature selection sizes. (**B**) Heatmap of 17-gene signature. (**C**) Comparison of AUC of four prediction models. (**D**) Comparison of accuracy, F1 score, precision, and recall of four prediction models. the abbreviations: naïve Bayes method (NB), neural network (NN), random forest (RF), and support vector machine (SVM).

Figure 1 presents an overview of the workflow of this study. Clinicopathological and gene expression features were ranked using five ranking-based feature selection methods. The feature selection procedure was performed independently of the four machine learning methods. Using the five ranking-based feature selection methods, 770 genes were listed in the highest order of each feature. Machine learning analysis was performed on the 17 highest genes in each feature in order to select the features that best predict brain metastasis among the five ranking-based feature selection methods. For each feature, four areas under the curves (AUCs) were derived by four machine learning algorithms, and the feature with the highest sum of the four AUC values was selected (Supplementary Table S1). The AUC is an effective way to indicate the diagnostic accuracy of a test. AUC values range from 0 to 1, where 0 represents a perfectly inaccurate test and 1 represents a perfectly accurate test. In general, AUC values are interpreted as follows: 0.5 (no discrimination), 0.7–0.8 (acceptable), 0.8–0.9 (excellent), and >0.9 (outstanding) [38]. Among the five ranking-based feature selection methods, chi-square had the highest AUC. We used the chi-square method as a ranking-based feature selection method to reduce the feature dimensions. A previous gene expression profile study also confirmed that the chi-square-based gene selection method could improve the performance of prediction models [39]. To determine the optimal number of relevant features, we compared the AUC of the four machine learning methods for feature sizes between 2 and 57 (Figure 2A). Among feature sizes between 2 and 57, 17 features had the highest sum of AUC. Clinicopathological and gene expression features were ranked using the chi-square method. The 17 features are summarized in Table 1. Among these 17 features, there were no clinicopathological factors; 11 genes (COMP, MEG3, ITGA11, COL1A1, FBN1, NR4A3, DCN, PDPN, CYP1B1, SPARC, and SRPX2) were upregulated during BM, and six genes (SMC3, ERMP1, BTG1, SORD, ARHGAP32, and SNRPF) were downregulated. Of the 17 features, there were 11 ECM-remodeling-related genes (SPARC, SMC3, COL1A1, SRPX2, FBN1, MEG3, COMP, ITGA11, PDPN, NR4A3, and DCN), 7 EMT-related genes (SORD, ARHGAP32, SNRPF, COL1A1, FBN1, MEG3, and DCN), and 4 angiogenesis-related genes (SRPX2, BTG1, ERMP1, and CYP1B1). Figure 2B shows a heatmap of the unsupervised clustering analyses of the 17 genes. Eleven upregulated genes were highly expressed in the group with BM, and six downregulated genes were slightly expressed. In Figure 2A, 17 features show the highest AUC values in NB, RF, and SVM. However, regarding the NN value, 57 features have a higher AUC value than 17 features (0.842 vs. 0.839). Therefore, based on the AUC value, the optimal number of features is 17 features in NB, RF, and SVM, and 57 features in NN. However, the difference in AUC between 17 features and 57 features in NN is not large, and, for consistency of analysis, 17 features were also used for the analysis in NN. In Figure 2C, four machine learning algorithms were analyzed with 17 features. The stratified k-fold validation receiver operating characteristic (ROC) curve for RF exhibited the highest AUC value of 0.858 (Figure 2C). The remaining three models also showed similar AUC values (NB: 0.845, NN: 0.839, and SVM: 0.849). The accuracy, F1 score, precision, and recall values ranged from 0.75 to 0.78 (Figure 2D). These results indicate that the mRNA expression profile consisting of the 17 genes is valuable for predicting BM in LUAD.

**Figure 3:**
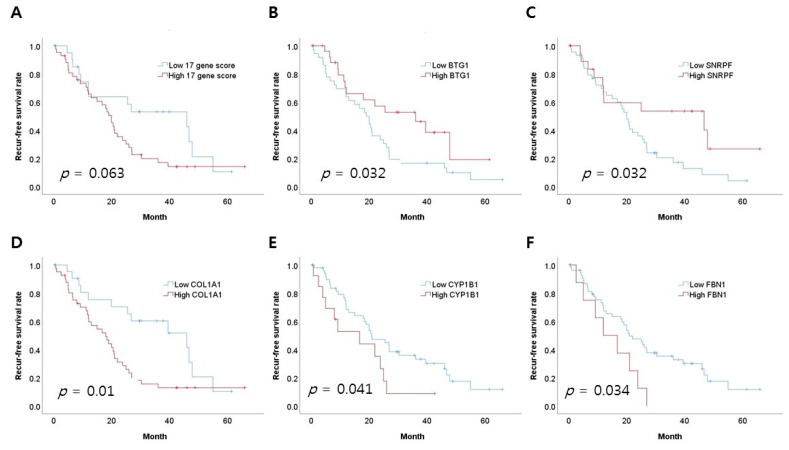
Comparison of survival rates according to 17 genes. (**A**) Recurrence-free survival (RFS) and 17-gene score. (**B**) RFS and BTG1. (**C**) RFS and SNRPF. (**D**) RFS and COL1A1. (**E**) RFS and CYP1B1. (**F**) RFS and FBN1.

We performed a survival analysis of 17 genes using normalized NanoString data. The calculation of the 17-gene score was defined as follows: 11 upregulated genes—6 downregulated genes. In the receiver operating characteristic (ROC) curves, the value representing the maximum joint sensitivity and specificity was determined as the cutoff. The cutoff values of the 17-gene score were defined as follows: SPARC (9483), SORD (495), COL1A1 (11831), SMC3 (524), ARHGAP32 (213), SNRPF (607), SRPX2 (78), BTG1 (1450), ERMP1 (211), FBN1 (1715), MEG3 (193), COMP (553), ITGA11 (362), PDPN (71), CYP1B1 (345), NR4A3 (52), and DCN (629). Patients with a high 17-gene score tended to have a lower recurrence-free survival (RFS) than patients with a low 17-gene score, but were not statistically significant (*p* = 0.063, Figure 3A). High BTG1 and SNRPF mRNA expressions were correlated with a favorable RFS rate (*p* = 0.032, Figure 3B; and *p* = 0.032, Figure 3C). High COL1A1, CYP1B1, and FBN1 mRNA expressions were correlated with a worse RFS rate (*p* = 0.01, Figure 3D; *p* = 0.041, Figure 3E; and *p* = 0.034, Figure 3F, respectively). Other genes did not correlate with an RFS rate. There was no difference in the overall survival (OS) rate between patients with a high 17-gene score and patients with a low 17-gene score (*p* = 0.612, Supplementary Figure S1A). A high CYP1B1 mRNA expression was correlated with a worse OS rate (*p* = 0.049, Supplementary Figure S1B). Other genes did not correlate with the OS rate.

### 2.2. Relationship between the 17-Gene Signature and TNM Stage

**Figure 4:**
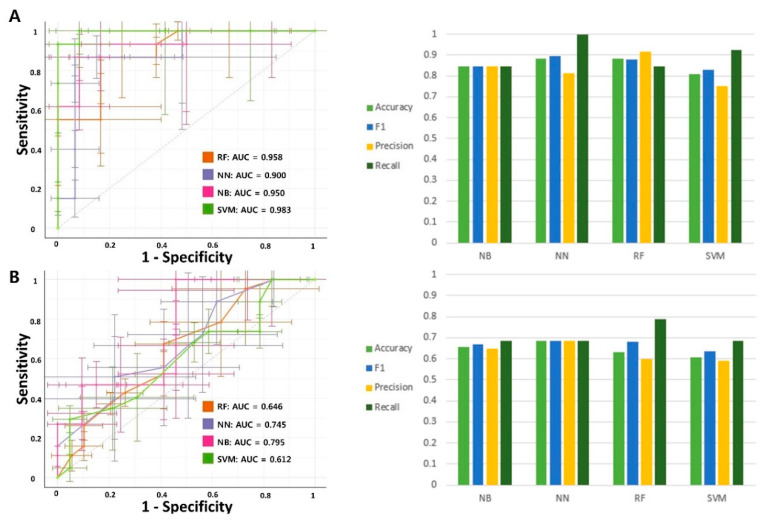
Predictive model building for brain metastasis in lung adenocarcinoma according to TNM stage. (**A**) Comparison of AUC, accuracy, F1 score, precision, and recall of four prediction models in TNM stage I or II. (**B**) Comparison of AUC, accuracy, F1 score, precision, and recall of four prediction models in TNM stage III. the abbreviations: naïve Bayes method (NB), neural network (NN), random forest (RF), and support vector machine (SVM).

As tumor aggression is different for each tumor stage, the expression of BM-related genes or BM prediction may be different for each tumor stage. We performed subgroup analysis according to the TNM stage. The number of patients with TNM stage I or II was lower than that of patients with stage III; thus, the data from patients with stage I and II were combined and analyzed. In stage I or II (*n* = 26), three models showed very high predictive power, with an AUC value of 0.9 or higher (NB 0.95, NN 0.9, RF 0.958, and SVM 0.983) (Figure 4A). The accuracy, F1 scores, precision, and recall values for the four models ranged from 0.75 to 1. However, in stage III (*n* = 38), the AUC value was lower than that in stage I or II (NB 0.795, NN 0.745, RF 0.646, and SVM 0.612) (Figure 4B). The accuracy, F1 score, precision, and recall values ranged from 0.59 to 0.78 for the four models. These results show that the 17-gene signature predicts BM well in the early stage, but its predictive power decreases in the late stage.

Postoperative extracranial metastasis may occur in patients with or without BM, and this may serve as a confounding factor in predicting BM. Postoperative extracranial metastasis was found in 15 (46.9%) of 32 patients with BM, and in 14 (43.8%) of 32 patients without BM (Supplementary Table S2). There was no statistical difference in the frequency of extracranial metastases between the group without BM and those with BM (*p* > 0.999). If the frequency of extracranial metastases varies greatly depending on the TNM stage, it may also affect the prediction of BM. In patients without BM, extracranial metastasis was found in 5 of 13 TNM stage I or II patients (38.5%), and in 9 of 19 TNM stage III patients (47.4%). The frequency of extracranial metastases was slightly higher in TNM stage III than in TNM stage I or II, but it was not statistically significant (*p* = 0.725). In patients with BM, extracranial metastasis was found in 7 of 13 TNM stage I or II patients (53.8%), and in 8 of 19 TNM stage III patients (42.1%). The frequency of extracranial metastases was slightly higher in TNM stage I or II than in TNM stage III, but it was not statistically significant (*p* = 0.72).

### 2.3. Principal Component Analysis and Pathway Enrichment Analysis

**Figure 5:**
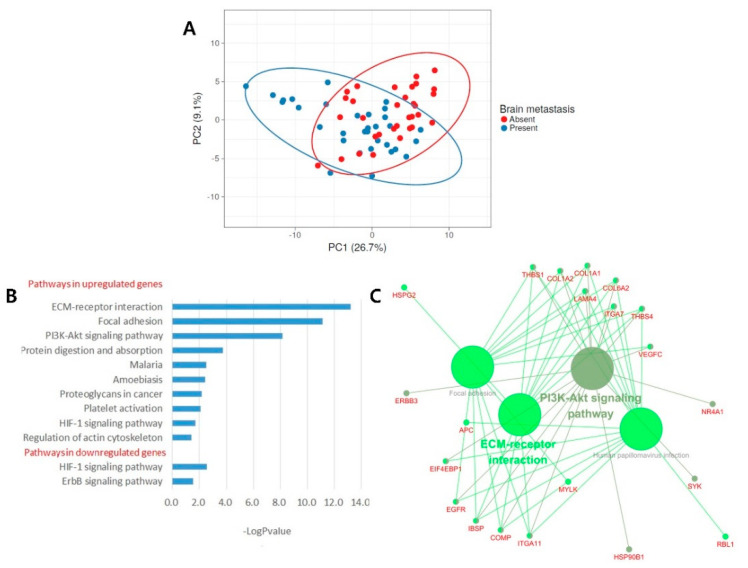
Pathway enrichment analysis for BM-related genes. (**A**) Principal component analysis of differentially expressed genes. (**B**) KEGG pathway terms related to brain metastasis in lung adenocarcinoma. (**C**) The gene network analysis related to brain metastasis in lung adenocarcinoma.

To identify the functions of BM-related genes, we identified 116 genes that were significantly associated with BM, with a *p*-value of <0.05 and a false discovery rate (FDR) of <0.25 (Supplementary Table S3). There were 86 upregulated genes and 30 downregulated genes. Supplementary Figure S2 shows a heatmap of the unsupervised clustering analyses of the 116 genes: 86 upregulated genes were highly expressed in the BM group, and 30 downregulated genes were slightly expressed. A principal component analysis revealed that the first principal component (PC1) explained 26.7% of the variance, and the second principal component (PC2) explained 9.1% (Figure 5A). Pathway enrichment analyses were performed using 86 and 30 genes to identify the pathways leading to BM. In the KEGG pathway analysis, 10 pathways were identified (*p*-value < 0.05) in 86 upregulated genes, and two pathways were identified, with *p*-values of <0.05, in 30 downregulated genes (Figure 5B). Of a total of 12 pathways, 4 were with an FDR of <0.05, including the ECM–receptor interaction, focal adhesion, the PI3K–Akt signaling pathway, and protein digestion and absorption. The gene lists of these four pathways are summarized in Table 2. Of the four pathways, the ECM–receptor interaction showed the lowest FDR (FDR < 0.001), indicating that the ECM–receptor interaction contributed the most to BM. In the gene network analysis using ClueGO, genes of the ECM–receptor interaction node were found to be correlated with focal adhesion and the PI3K–Akt signaling pathway node (Figure 5C). Furthermore, as shown in Table 2, many genes overlapped between the ECM–receptor interaction, focal adhesion, and PI3K–Akt signaling pathways.

### 2.4. IHC Analysis

**Figure 6:**
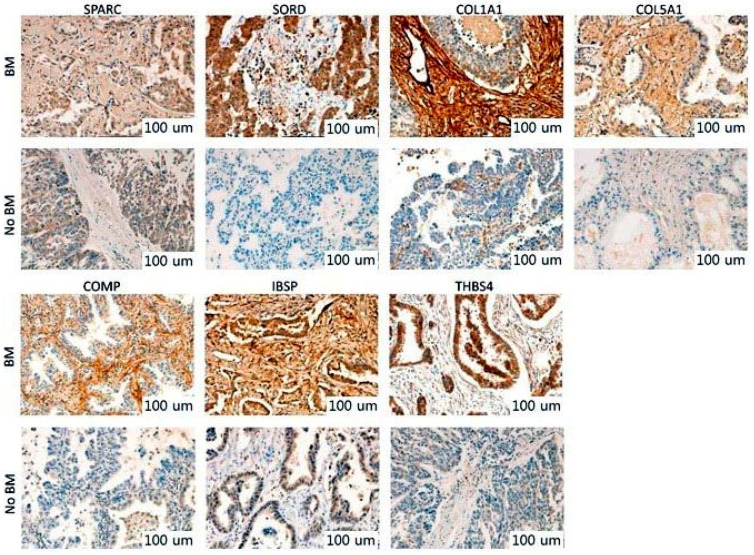
Immunohistochemistry analysis. Representative immunohistochemical images of SPARC, SORD, COL1A1, COL5A1, COMP, IBSP, and THBS4.

**Figure 7:**
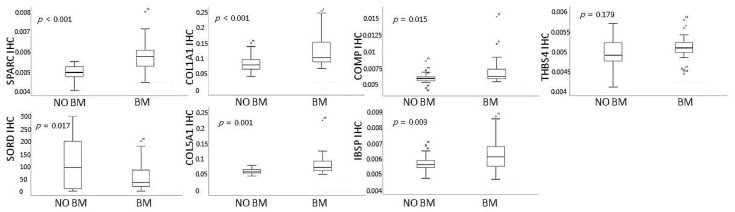
Immunohistochemistry analysis. Comparison of protein expression of SPARC, SORD, COL1A1, COL5A1, COMP, IBSP, and THBS4 between lung adenocarcinoma with brain metastasis and lung adenocarcinoma without brain metastasis. Circles are outliers.

Pathway enrichment analysis revealed that ECM–receptor interaction was closely correlated with BM, and most of the 17 genes in the machine learning model were related to ECM remodeling. The top three genes (SPARC, SORD, and COL1A1) of the 17-gene expression signature from the machine learning analysis, and four genes (COL5A1, COMP, IBSP, and THBS4) with a high fold-change in the gene list of the ECM–receptor interaction pathway were verified by immunohistochemistry. Expression intensities of SPARC, COL1A1, COL5A1, COMP, IBSP, and THBS4 were interpreted in tumor stroma, as they were associated with ECM-related pathways, and the expression intensity of SORD was interpreted in tumor cells, as it was associated with the EMT pathway. Representative IHC expression levels of the seven genes in the groups with or without BM are summarized in Figure 6. In the tumor stroma, the IHC expression of SPARC and COL1A1 was significantly higher in the group with BM than that in the group without BM (*p* < 0.001 for both; Figure 7). In the tumor cell, the IHC expression of SORD was significantly lower in the group with BM than that in the group without BM (*p* = 0.017; Figure 7). In the tumor stroma, the IHC expression of COL5A1, COMP, and IBSP was significantly higher in the group with BM than that in the group without BM (*p* = 0.001 for COL5A1, *p* = 0.015 for COMP, and *p* = 0.003 for IBSP; Figure 7). However, the IHC expression of THBS4 was not correlated with BM (*p* = 0.179; Figure 7). Except for THBS4, the protein expression of the remaining six genes was consistent with the NanoString results. The AUCs of SPARC, SORD, COL1A1, COL5A1, COMP, and IBSP were 0.871, 0.686, 0.774, 0.793, 0.686, and 0.689, respectively (Supplementary Figure S3). In the multivariate logistic regression analysis of six genes, two genes were found to be independent predictors of BM (*p* = 0.004 for SPARC and *p* = 0.018 for COL5A1; Supplementary Table S4).

## 3. Materials and Methods

### 3.1. Clinical Samples

The study was approved by the Institutional Review Board of the Ajou University School of Medicine (AJIRB-BMR-KSP-18-374). The requirement for informed consent was waived, owing to the retrospective study design. The clinicopathological characteristics are summarized in Supplementary Table S5. Age, sex, smoking history, histologic subtype, and ALK translocation were not associated with BM. Of 655 patients with LUAD who underwent lung resection at Ajou University Hospital, BM was found in 45 patients (6%) during follow-up. Of the 45 patients with BM, 32 were randomly selected. The higher the TNM stage, the more aggressive the tumor is, and the easier it is for BM to be triggered. We randomly selected TNM stage-matched LUAD samples (32 cases) that did not develop BM during the follow-up period. None of the patients had BM at the time of preoperative radiological findings. During the follow-up period, we attempted to identify BM using a brain CT or MRI scan. The median follow-up time was 35.81 months (range: 4–82 months). Pathological tumor staging was determined according to the eighth edition of the TNM classification.

### 3.2. Gene Expression Analysis Using the NanoString nCounter Assay

A total of 770 ECM-remodeling-related, EMT-related, and angiogenesis-pathway-related genes were used for the NanoString nCounter Assay (NanoString nCounter PanCancer Progression panel; NanoString Technologies, Seattle, WA, USA) [63]. The 770 genes included 277 genes related to angiogenesis, 254 genes related to ECM, and 269 genes related to EMT. The reporter code and capture probe sets were mixed with the total RNA. After the hybridization reaction, the sample was transferred to the preparation station and a high-sensitivity procedure was performed. After scanning the sample using the nCounter Digital Analyzer (NanoString Technologies Inc., Seattle, WA, USA), normalization was performed using the geometric mean of the positive control counts and a housekeeping gene.

### 3.3. IHC Staining

We constructed a tissue microarray with two cores, 2 mm in diameter. IHC was performed using a Benchmark XT automatic IHC staining device with an OptiView DAB IHC Detection Kit (Ventana Medical Systems, Tucson, AZ, USA). The experimental information for IHC is summarized in Supplementary Table S6. We measured the intensity of IHC staining in tumor stroma for genes related to the ECM remodeling pathway, and the intensity of IHC staining in tumor cells for genes related to the EMT pathway. For tumor stroma, we measured the intensity of IHC staining using the scientific image analysis software, ImageJ [64]. In the micrograph at 200× magnification, the intensity was measured at three locations in the tumor stroma, and then the average value was obtained. For tumor cells, we used H-scores to interpret genes related to the EMT pathway [65]. For the H-score, we evaluated the intensity of protein on a four-point intensity scale: 0 (no staining), 1 (light yellow = faint staining), 2 (yellow-brown = moderate staining), and 3 (brown = strong staining). We also evaluated the percentages of positive cells (0–100%). The H-scores (0–300) were calculated by multiplying the percentage of cells by the intensity score.

### 3.4. Machine Learning Approach and Statistical Analysis

For modeling approaches, four different machine learning algorithms for binary classification, including SVM, RF, NN, and NB, were applied using Orange version 3.27 software (Bioinformatics Laboratory at the University of Ljubljana, Ljubljana, Slovenia) [66]. Orange version 3.27 software used sklearn.feature_selection for ranking-based feature selection methods. We performed SVM analysis using RBF kernel (C = 1.0 and gamma = ‘auto’), RF analysis using 10 trees, and NN analysis using multi-layer perceptron architecture (activation function = ReLu, neurons per hidden layer = 100, Adam optimization and maximal number of iterations = 200). A total of five clinicopathological features (sex, age, micropapillary pattern, solid pattern, and smoking history) and 770 gene features were used to predict BM using a machine learning approach. Feature reduction and selection methods were used to increase the prediction accuracy. Among the five ranking-based feature selection methods (information gain, information gain ratio, Gini decrease, chi-square, and ReliefF), the method with the highest AUC value was selected. Among the feature sizes of between 2 and 57, the feature size with the highest AUC value was selected to determine the optimal number of relevant features. To evaluate the performance of predictive classification models, we used a stratified k-fold cross-validation. We used a stratified k-fold cross-validation as the data splitting method. The K value was set to 3. The aim of 3-fold cross-validation is to divide the data into three groups, extract one of the groups, and use it as a validation set (*n* = 21). The remaining two groups are used as a training set (*n* = 43). This process is then repeated three times. The three results can then be averaged to produce a final result. To compare the performance of the predictive model, the ROC was drawn, and the AUCs were calculated. We calculated five performance measures: AUC, accuracy (the rate of correct classification), F1 score (the harmonic mean of the model’s precision and recall), precision (positive predictive value), and recall (sensitivity).

NanoString nSolver analysis software (NanoString Technologies Inc., Seattle, WA, USA) was used to obtain normalized data, fold changes, and *p*-values. The “fdrtool” package in R (The R Foundation, Vienna, Austria) was used to calculate the false discovery rate (FDR). A *t*-test was used to compare the continuous values. In the ROC curves of the IHC data, the value representing the maximum joint sensitivity and specificity was determined as the cutoff. The probability of BM, based on IHC expression profiles, was investigated with multivariate logistic regression analyses using the forward conditional method. IBM SPSS Statistics 25 software (IBM, Armonk, NY, USA) or R version 3.5.3 (The R Foundation) was used for the analyses, and a *p*-value < 0.05 was considered statistically significant. A gene expression heatmap and principal component analysis were created using the ClustVis software [67]. For pathway enrichment analysis, we used the DAVID Bioinformatics Resources 6.8 tool [68]. DAVID is a free online bioinformatics resource developed by the Laboratory of Immunopathogenesis and Bioinformatics. We performed a KEGG pathway analysis using the DAVID Bioinformatics Resources 6.8 tool. Currently, the KEGG pathway includes a total of 548 pathways. In the KEGG pathway analysis, the *p*-value cutoff was set to 0.05. We used the FDR method for multiple hypothesis testing. The Cytoscape (ClueGO) plug-in [69] was used for the gene network analysis.

## 4. Discussion

In this study, we made several important discoveries regarding the LUAD BM. First, we identified a 17-gene expression signature that could predict BM before BM occurred in LUAD. Most of the 17 genes were associated with ECM remodeling. The 17-gene expression signature showed a higher BM predictive ability in early-stage LUAD than that in late-stage LUAD. Second, the ECM–receptor interaction pathway was significantly associated with BM, as assessed through KEGG pathway enrichment analysis. Third, the protein expression of the major genes in the 17-gene expression signature was also closely related to BM, as revealed by IHC. For practical use, the NanoString method is expensive, requires many samples, and is not user-friendly. However, in pathology laboratories, IHC is inexpensive and easy to set up.

As revealed by subgroup analysis, the 17-gene expression signature of early-stage LUAD showed a greater ability to predict BM than that of late-stage LUAD. As a tumor progresses, the ECM remodeling, EMT, and angiogenesis pathways are activated for invasion and metastasis [5,9,14]. Therefore, because most of the late-stage LUADs express genes related to ECM remodeling, the difference in expression of genes related to ECM remodeling between the groups with and without BM cannot be significant.

KEGG pathway enrichment analysis revealed that ECM–receptor interaction, focal adhesion, the PI3K–Akt signaling pathway, and the protein digestion and absorption pathways were correlated with BM. Most genes involved in focal adhesion, the PI3K–Akt signaling pathway, and the protein digestion and absorption pathways overlap with genes in the ECM–receptor interaction pathway (12/15 for focal adhesion, 12/15 for the PI3K–Akt signaling pathway, and 5/6 for the protein digestion and absorption pathways). As the rest of the KEGG pathways are also related to the ECM–receptor interaction pathway, the ECM–receptor interaction pathway plays a very important role in BM.

In our gene network analysis, genes of the ECM–receptor interaction node were closely correlated with focal adhesion and the PI3K–Akt signaling pathway. Focal adhesion is a subcellular structure between the cell and the ECM. Focal adhesion plays an important role in cell migration through tissues. Focal adhesion kinase directly activates focal adhesion signaling pathways and promotes tumor metastasis through effects on malignant cells [70]. The PI3K–Akt signaling pathway is also known to induce cell proliferation and metastasis. PI3K/AKT pathway inhibitor reversed focal adhesion switching and inhibited cancer cell motility in esophageal squamous cell carcinoma [71]. MUC15, a subtype of the mucin family, can suppress tumor metastasis by inhibiting PI3K/AKT signaling in renal cell carcinoma [72]. TFAP4 also promotes metastasis of hepatocellular carcinoma by activating PI3K/AKT signaling pathway [73].

In previous studies, it was reported that the genes belonging to our 17-gene expression signature were associated with metastasis. The expression of SPARC and DCN is significantly higher in prostate cancer cell lines that actively invade the astrocyte monolayer [74]. In craniopharyngioma, a higher tumor stroma expression of SPARC leads to higher brain infiltration [75]. These results suggest that SPARC can induce brain infiltration during BM. Bao et al. reported that SPARC was a key mediator of the TGF-β signaling pathway and promoted invasion and metastasis of renal cell carcinoma [76]. In lung squamous cell carcinoma, COL1A1 overexpression in the microenvironment is highly correlated with lymph node metastasis [77]. SRPX2 promotes cell proliferation and metastasis in ESCC cells [78]. FBN1 promotes ovarian cancer metastasis via the p53- and SLUG-associated signaling [79]. COMP promotes metastasis and invasion of colorectal cancer by activating the EMT pathway [80]. An overexpression of microenvironmental ITGA11 is associated with a high tumor grade and poor prognosis in breast cancer [81]. High PDPN protein expression is significantly correlated with lung metastasis in patients with osteosarcoma [82]. KEGG pathway enrichment analysis showed that the ECM–receptor interaction pathway included 13 genes that were correlated with BM. In previous studies, it was reported that the 13 genes of the ECM–receptor interaction pathway were associated with metastasis. LAMA4 upregulation promotes hepatic metastasis in pancreatic cancers [83]. THBS1 induces hepatic metastasis by enhancing EMT in colorectal cancer [84]. THBS4 upregulation promotes the proliferation and metastasis of HCC [85]. COL5A1 is associated with the metastasis of LUAD [86]. IBSP overexpression is significantly related to lymph node metastasis in esophageal squamous cell carcinoma [87]. A high expression of COL5A2 is associated with metastasis in renal cell carcinoma [88]. ITGA7 upregulation promotes the proliferation and invasion of breast cancer cells [89].

In MEG3, COMP, and ITGA11, in Table 1, the fold change was 2 or more, but the χ² value was 7.041. In SPARC and SORD, the fold change was less than 2, but the χ² value was 9.375. Therefore, even if the fold change is high, the χ² value may be low. The fold change and *t*-test were widely used in the differential gene expression analysis. However, differential gene expression analysis using fold change and *t*-test also has some drawbacks. When using fold change, genes with a high reproducibility but small differences in relative expression values can be ignored because they do not take into account measurement error (variance) [90]. The *t*-test can be criticized for requiring a specific distribution assumption [90]. There is no clear criterion for the choice of thresholds for fold change and *p*-value. Fold change and *p*-value cutoffs can also significantly alter microarray interpretations [91]. Furthermore, the accuracy of predicting a cancer prognosis has improved by 15–20% over the past few years by applying machine learning (ML) technology [92].

A 17-gene signature was derived using the chi-square method, which predicted brain metastasis well. Through a KEGG pathway analysis, we demonstrated that 13 ECM–receptor interaction pathway genes are associated with brain metastasis. Three genes (COL1A1, COMP, and ITGA11) overlap between the 17-gene signature and the 13 ECM–receptor interaction pathway genes. The 17-gene signature plays a more important role for BM prediction. The 13 ECM–receptor interaction pathway genes suggest that ECM remodeling plays an important role during BM. The protein expression of the top three genes of the 17-gene signature and four genes among the 13 ECM–receptor interaction pathway genes showed similar results to those of the NanoString. These results indicate that both gene sets are involved in BM.

We used four machine learning classifiers (NB, NN, RF, and SVM) to determine whether gene expression could predict BM. AUC, accuracy, F1 score, precision, and recall were compared using four machine learning classifiers. Because all four machine learning classifiers have been used in many gene expression studies [93,94,95,96], it is difficult to know which classifier is suitable. Therefore, we used all four widely used machine learning classifiers. All four classifiers have an AUC value of 0.8 or more and an accuracy of 0.7 or more, indicating that our 17-gene signature can predict BM relatively well.

Our study has several limitations. First, the genes involved in ECM remodeling were expressed in the tumor microenvironment. However, NanoString analysis cannot measure gene expression by distinguishing between the tumor cells and the tumor microenvironment. According to our IHC results, the genes involved in ECM remodeling were more strongly expressed in the tumor microenvironment of LUAD with BM than in LUAD without BM. Therefore, even in NanoString analysis, the tumor microenvironment may have a greater influence on the expression of genes involved in ECM remodeling than the tumor cells. Second, despite the relatively small sample size, our results were not verified in an external validation set. Therefore, in the future, our findings need to be validated by external studies with larger sample sizes. Third, postoperative extracranial metastasis was found in 43% of patients without BM and in 46% of patients with BM. Postoperative extracranial metastasis may serve as a confounding factor in predicting BM. It is possible that the gene signature we found is not specific for BM and may be affected by other extracranial metastases. It is difficult to find very small-sized brain metastases on a radiological examination. Therefore, the possibility that a very small-sized BM patient was included in the group without BM in this study cannot be excluded.

## 5. Conclusions

We discovered a novel tumor nonimmune-microenvironment-related signature related to LUAD BM that provides insight into the biological mechanisms involved in BM development in LUAD. Our results can assist with predicting those patients who are likely to develop LUAD BM, in advance, and make treatment decisions.

## Figures and Tables

**Table 1:** Feature ranking of 770 Pan-Cancer progression panel genes.

Rank	Features	χ² Value	Fold Change	Function	Reference
1	SPARC	9.375	1.67	ECM remodeling	[40]
2	SORD	9.375	−1.54	EMT	[41]
3	COL1A1	8.166	1.87	ECM remodeling, EMT	[42,43]
4	SMC3	8.166	−1.27	ECM remodeling	[44]
5	ARHGAP32	8.166	−1.61	EMT	[45]
6	SNRPF	8.166	−1.61	EMT	[46]
7	SRPX2	8.166	1.6	ECM remodeling, angiogenesis	[47,48]
8	BTG1	8.166	−1.38	Angiogenesis	[49]
9	ERMP1	7.041	−1.38	Angiogenesis	[50]
10	FBN1	7.041	1.85	ECM remodeling, EMT	[51,52]
11	MEG3	7.041	2.56	ECM remodeling, EMT	[53,54]
12	COMP	7.041	2.63	ECM remodeling	[55]
13	ITGA11	7.041	2.03	ECM remodeling	[56]
14	PDPN	7.041	1.71	ECM remodeling	[57]
15	CYP1B1	7.041	1.71	EMT, angiogenesis	[58,59]
16	NR4A3	7.041	1.8	ECM remodeling	[60]
17	DCN	7.041	1.73	ECM remodeling, EMT	[61,62]

ECM, extracellular matrix; EMT, epithelial–mesenchymal transition.

**Table 2:** Four KEGG pathway gene lists (FDR < 0.05).

ECM–Receptor Interaction
LAMA4, HSPG2, THBS1, THBS4, COL1A1, COMP, COL1A2, COL5A1, IBSP, COL6A2, COL5A2, ITGA11, ITGA7
Focal adhesion
LAMA4, VEGFC, THBS1, EGFR, THBS4, MYLK, COL1A1, COMP, COL1A2, COL5A1, IBSP, COL6A2, COL5A2, ITGA11, ITGA7
PI3K–Akt signaling pathway
LAMA4, VEGFC, THBS1, EGFR, THBS4, COL1A1, COMP, NR4A1, COL1A2, COL5A1, IBSP, COL6A2, COL5A2, ITGA11, ITGA7
Protein digestion and absorption
COL1A1, COL18A1, COL1A2, COL5A1, COL6A2, COL5A2

## Data Availability

The data presented in this study are available on request from the corresponding author. The data are not publicly available due to ethical considerations.

## Supplementary Materials

The following are available online at https://www.mdpi.com/article/10.3390/cancers13174468/s1, Figure S1: Overall survival rates and 17-gene score (A) and overall survival rates and CYP1B1 (B); Figure S2: Heatmap of differentially expressed genes; Figure S3: Comparison of AUC according to SPARC, SORD, COL1A1, COL5A1, COMP, and IBSP protein expression; Table S1: Ranking-based feature selection methods; Table S2: Extracranial metastasis in group without brain metastasis and group with brain metastasis; Table S3: 116 genes with elevated or reduced expression in the brain metastasis group (*p*-value < 0.05); Table S4: Multivariate logistic analyses for brain metastasis; Table S5: Demographic and clinical characteristics of patients; Table S6: Information on the used antibodies for immunohistochemistry.
