# Supplementary material for: Tumor Nonimmune-Microenvironment-Related Gene Expression Signature Predicts Brain Metastasis in Lung Adenocarcinoma Patients after Surgery: A Machine Learning Approach Using Gene Expression Profiling
Source: Cancers (Basel). 2021 Sep 5;13(17):4468. doi: 10.3390/cancers13174468 (PMC8430997; doi:10.3390/cancers13174468)
Supplement: Supplementary file 1 [file cancers-13-04468-s001.zip › cancers-1302790-supplementary.pdf]

cancers

MDPI

Tumor Nonimmune-Microenvironment-Related Gene Expression Signature Predicts Brain Metastasis in Lung Adenocarcinoma Patients after Surgery: A Machine Learning Approach Using Gene Expression Profiling

Seokjin Haam, Jae-Ho Han, Hyun Woo Lee and Young Wha Koh

Supplementary Materials

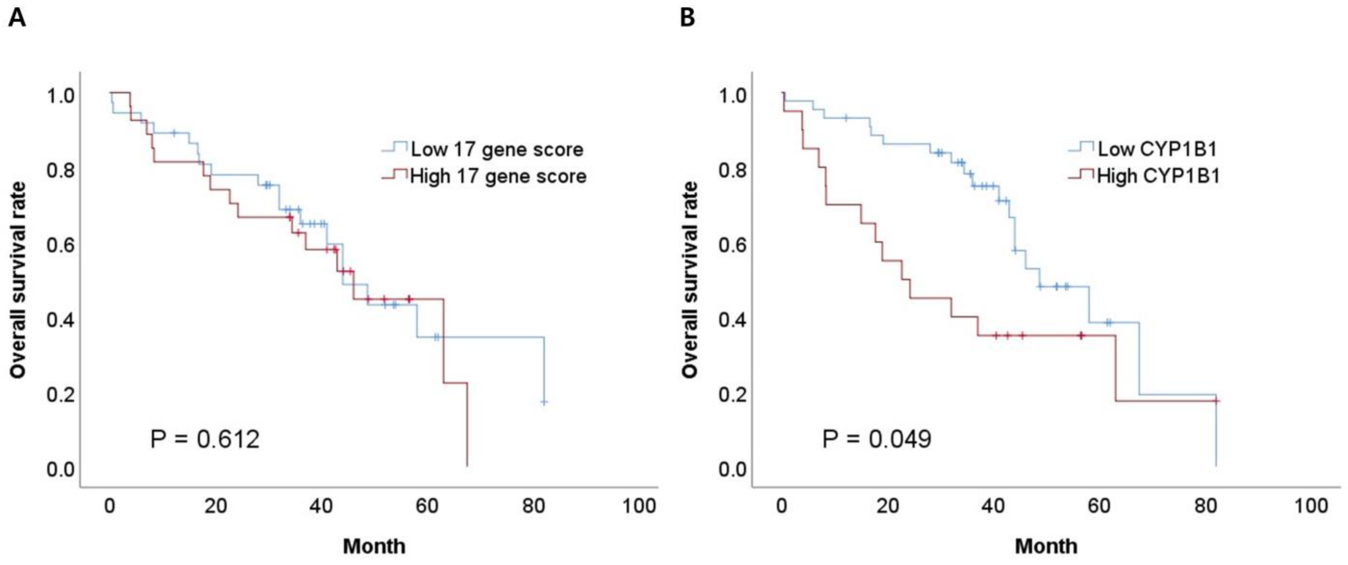

Figure S1. Overall survival rates and 17-gene score (**A**) and overall survival rates and CYP1B1 (**B**).

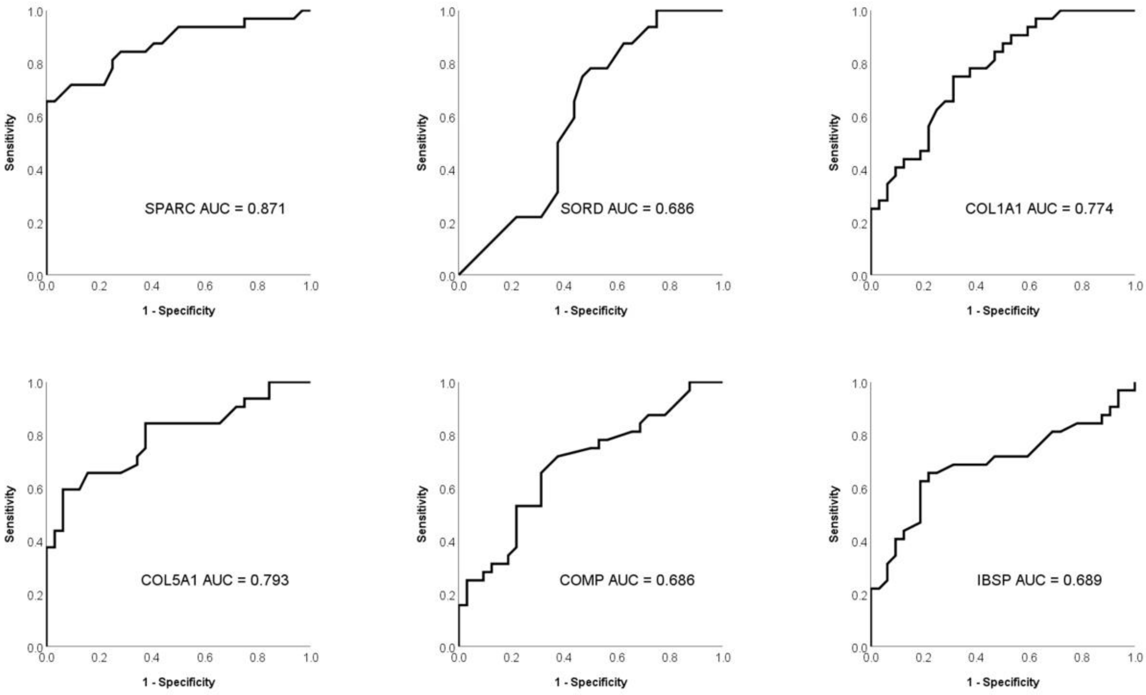

Figure S3. Comparison of AUC according to SPARC, SORD, COL1A1, COL5A1, COMP, and IBSP protein expression.

Table S1. Ranking-based feature selection methods.

Method	SVM AUC	RF AUC	NN AUC	NB AUC	Sum of AUC
Information gain (17 feature)	0.737	0.807	0.729	0.906	3.179
Information gain ratio (17 feature)	0.747	0.790	0.736	0.906	3.179
Gini decrease (17 feature)	0.747	0.821	0.731	0.905	3.204
Chi-square (17 feature)	0.849	0.858	0.839	0.845	3.391
ReliefF (17 feature)	0.843	0.749	0.857	0.798	3.247

Table S2. Extracranial metastasis in group without brain metastasis and group with brain metastasis.

Extracranial metastasis	Brain metastasis positive	Brain metastasis negative	*p* value
Absent	17 (53.1%)	18 (56.3%)	> 0.999
Present	15 (46.9%)	14 (43.8%)	

Table S3. 116 genes with elevated or reduced expression in the brain metastasis group (p value < 0.05).

86 elevated genes
ACVRL1, ADAMTS12, AEBP1, APC, APOD, APOE, APOH, ASPN, BNC2, CALD1, CAMK2A, CAMK2B, CAMP, CCL8, CCR3, CDH11, CDK14, COL18A1, COL1A1, COL1A2, COL5A1, COL5A2, COL6A2, COMP, CSPG4, CTSK, CYP1B1, DCN, DPYSL3, ECM2, EDN1, EGFL7, EGFR, FAP, FBN1, FMOD, FOXC2, FSTL1, GDF6, HAS1, HSPG2, IBSP, ICAM1, IGFBP4, IGFBP7, IL1RL1, ISLR, ITGA11, ITGA7, JAM2, KIAA1462, LAMA4, MEG3, MMP13, MMP17, MMP24, MYLK, NAP1L3, NR4A1, NR4A3, OGN, OLFML2B, PCOLCE, PDPN, PLXNC1, PMP22, PRELP, PRSS8,

PYCARD, RUNX1T1, SERPINH1, SFRP2, SNAI2, SPARC, SRPX2, TAL1, TCF4, THBS1, THBS4, THY1, TIMP2, TNFRSF12A, TXNIP, VEGFC, ZCCHC24, ZEB1
30 reduced genes
ARHGAP32, BTG1, CBLC, EIF4EBP1, ERBB3, ERMP1, GRHL2, HIPK2, HK2, HSP90B1, MED1, NCL, NME1, NRCAM, PDCD10, POPDC3, PROM1, RBL1, RPS27A, SACS, SMAD3, SMC3, SNRPF, SORD, ST14, SYK, TCEB1, TF, TJP2, TOM1L1

Table S4. multivariate logistic analyses for brain metastasis.

	**OR**	**95%CI**	***p*-value†**
SPARC	38.78	3.246-463.6	0.004
SORD	0.305	0.030-3.146	0.319
COL1A1	8.351	0.999-69.83	0.051
COL5A1	8.566	1.436-51.09	0.018
COMP	5.499	0.751-40.28	0.093
IBSP	4.689	0.549-40.03	0.158

Table S5. Demographic and clinical characteristics of patients.

**Variable**	**Brain metastasis, number (%)**	**No brain metastasis, number (%)**	***p* value**
Age, median (range) (years)	57.5 (35–78)	65.5 (47–86)	0.082
Male sex	22 (68.8%)	19 (59.4%)	0.603
Smoking history	11 (39.3%)	11 (36.7%)	>0.999
Pathologic TNM stage			
I	8 (25%)	8 (25%)	
II	5 (15.6%)	5 (15.6%)	
III	19 (59.4%)	19 (59.4%)	
Histologic subtype			0.236
Acinar	14 (43.8%)	10 (31.3%)	
Papillary	5 (15.6%)	5 (15.6%)	
Lepidic	0 (0%)	4 (12.5%)	
Micropapillary	3 (9.4%)	1 (3.1%)	
Solid	10 (31.3%)	12 (37.5%)	
ALK translocation	1 (3.1%)	0 (0%)	>0.999

Smoking history was collected for 28 patients for group with brain metastasis and 30 patients for group without brain metastasis.

Table S6. Information on the used antibodies for immunohistochemistry.

**Name**	**Clonality**	**Clone name**	**Catalog name**	**Dilution**	**Manufacturer**
SPARC	Rabbit polyclonal		ab203284	1:200	Abcam
SORD	Rabbit polyclonal		ab7046	1:200	Novus Biologicals
COL1A1	Rabbit polyclonal		ab34710	1:100	Abcam
COL5A1	Rabbit polyclonal		ab7046	1:50	Abcam
COMP	Rabbit polyclonal		ab231977	1:50	Abcam
IBSP	Rabbit polyclonal		ab52128	1:300	Abcam
THBS4	Rabbit polyclonal		S2121540	1:100	Mybiosource
