# Supplementary figures and images for: Tumor Nonimmune-Microenvironment-Related Gene Expression Signature Predicts Brain Metastasis in Lung Adenocarcinoma Patients after Surgery: A Machine Learning Approach Using Gene Expression Profiling
Source: Cancers (Basel). 2021 Sep 5;13(17):4468. doi: 10.3390/cancers13174468 (PMC8430997; doi:10.3390/cancers13174468)

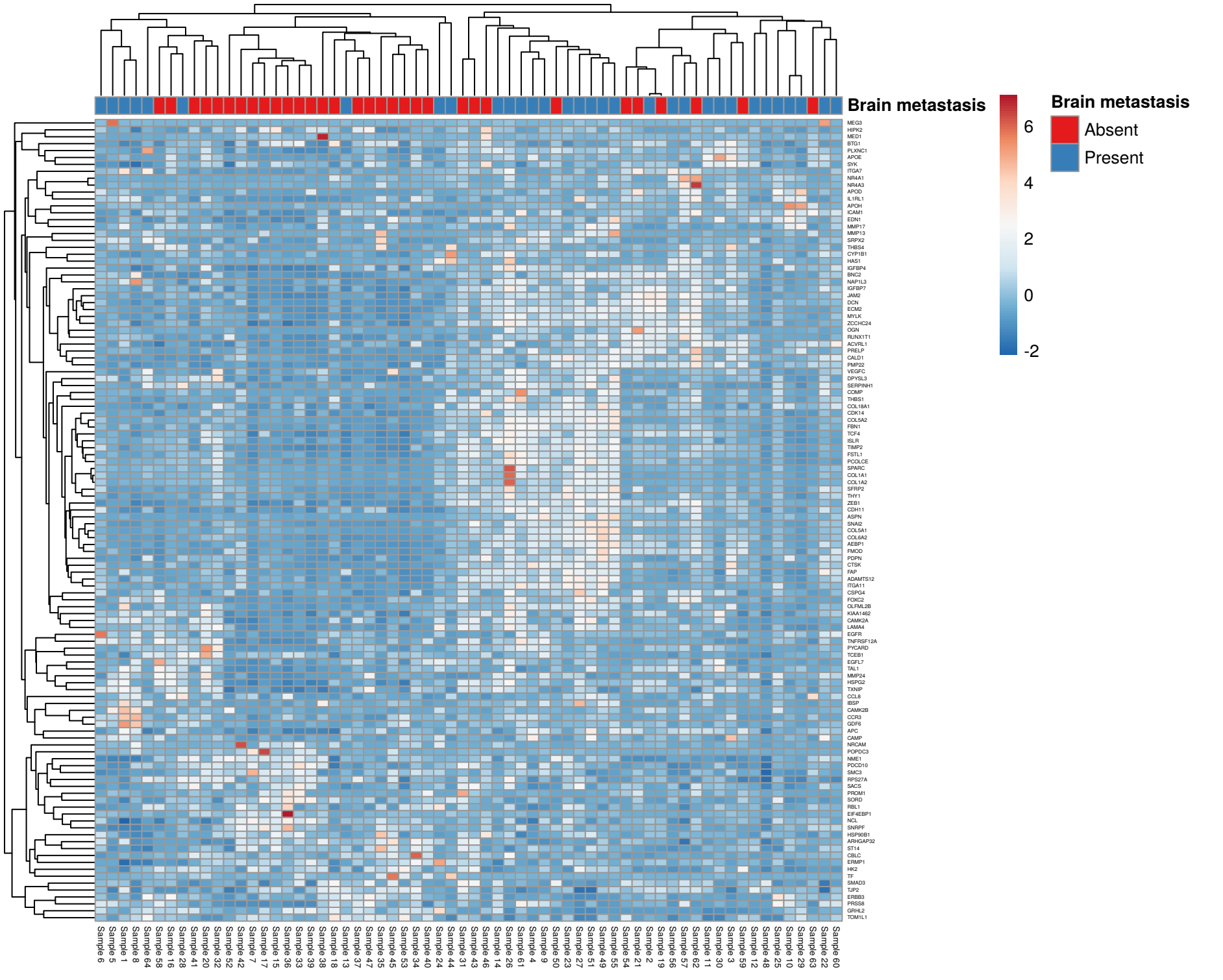

Supplement: Supplementary file 1 [file cancers-13-04468-s001.zip › Supplementary Figure 2.pdf]
